# Regional influences on community structure across the tropical-temperate divide

**DOI:** 10.1038/s41467-019-10253-6

**Published:** 2019-06-14

**Authors:** Alexander E. White, Kushal K. Dey, Dhananjai Mohan, Matthew Stephens, Trevor D. Price

**Affiliations:** 1 Department of Ecology and Evolution, University of Chicago, 1101 E 57th Street, Chicago, IL 60637 USA; 2 Data Science Lab, Office of the Chief Information Officer, Smithsonian Institution, 600 Maryland Avenue SW, Washington, 20024 DC USA; 3 National Museum of Natural History, Smithsonian Institution, MRC 166 PO Box 37012, Washington, DC 20013 USA; 4 Department of Statistics, University of Chicago, 5747 S Ellis Avenue, Chicago, IL 60637 USA; 5 Department of Epidemiology, Harvard University, 665 Huntington Avenue, Cambridge, MA 02115 USA; 6 Wildlife Institute of India, PO Box 18, Chandrabani, Dehradun 248001 India; 7 Department of Human Genetics, University of Chicago, 920 E 58th Street, Chicago, IL 60637 USA

**Keywords:** Biodiversity, Biogeography, Community ecology, Macroecology, Tropical ecology

## Abstract

Many models to explain the differences in the flora and fauna of tropical and temperate regions assume that whole clades are restricted to the tropics. We develop methods to assess the extent to which biotas are geographically discrete, and find that transition zones between regions occupied by tropical-associated or temperate-associated biotas are often narrow, suggesting a role for freezing temperatures in partitioning global biotas. Across the steepest tropical-temperate gradient in the world, that of the Himalaya, bird communities below and above the freezing line are largely populated by different tropical and temperate biotas with links to India and Southeast Asia, or to China respectively. The importance of the freezing line is retained when clades rather than species are considered, reflecting confinement of different clades to one or another climate zone. The reality of the sharp tropical-temperate boundary adds credence to the argument that exceptional species richness in the tropics reflects species accumulation over time, with limited transgressions of species and clades into the temperate.

## Abstract (web summary)

Multiple drivers maintain unique species assemblages at multiple biogeographic scales. Here, the authors show that the freezing line is a key barrier generating evolutionary differences in temperate and tropical bird communities across a steep elevational gradient in the Himalaya.

## Introduction

Discrete differences between the fauna and flora in different regions of the world have led biologists to delineate a nested hierarchy of biogeographic regions^1–4^. Starting with metrics of species turnover across space (beta diversity), Holt et al.^2^ identified 20 zoogeographic regions within which species are on average relatively closely related to each other, more so than between regions. These zoogeographic regions are nested within 11 larger realms, and due to strong barriers to dispersal imposed by oceans, boundaries of regions are often coincident with the boundaries of continents^5^. The long separation of different continents has therefore led to the more or less independent evolution of distinctive biotas (the collection of species that characterize the biogeographic region). However, other partitions between zoogeographic regions and realms have been drawn within continents, notably associated with tropical and temperate climates^2^. With no obvious barriers to dispersal, these realms must intergrade to some extent^6,7^. Indeed, a line delineating realms on continents may indicate sharp turnover in species composition or merely lie at a point of balance within a continuous gradient of compositional change^7^.

A long independent history of species accumulation in tropical and temperate climatic regimes (here defined as locations separated by the line of regular freezing) is implied if turnover between these realms is sharp, in much the same way that biotas on different continents have evolved independently. Such an independent history is integral to many models to explain differences in species composition and richness between communities in the tropics and the temperate^8–10^, yet evidence has been elusive. Evolutionary transitions in both plants^11^ and birds^12^ regularly occur across the tropical–temperate boundary, calling the historical discreteness of tropical and temperate biotas into question. Here, we introduce a method that ascribes species to biotas based on their geographical patterns of co-occurrence and subsequently assigns biotas to locations, crucially allowing multiple biotas to contribute to a single location. The method allows us to conceptually separate biotas (reflecting co-occurring species) from realms (where these biotas are found), ascertain the degree of intergradation between realms, and quantify the contribution of species to biotas and of biotas to both realms and local communities. Working with birds, we evaluate the extent to which realms intergrade, focusing on the tropical–temperate boundary. We combine these observations with a local study of this boundary by examining regional affinities of local communities across the steepest tropical–temperate gradient in the world, that of Himalaya.

Our methods extend Grade of Membership models^13^, which allow the units of analysis (e.g. species or geographic locations) to have partial membership in multiple clusters. These probabilistic models are used extensively in population genetics^14^ and Natural Language Processing^15^ and have also been applied in ecology^16^. By contrast, previous approaches to partitioning global diversity have used hard clustering techniques where by a locality can only belong to one realm^2–4,17^. In the Grade of Membership model, the species present at each location consist of a proportional mix of all biotas. The method achieves this by simultaneously inferring clusters of co-occurring species, which we term species motifs (equivalent to biotas), and the proportional contribution of each motif to a location; this inference therefore defines the biogeographic units (e.g. realms) and their inter-digitation. The motif contributions can be visualized on a map, identifying regions of mixture and patterns of turnover, thereby summarizing geographical structuring of the realms. We use the general term motif because the same analytical framework can be applied to other features of biodiversity, including whole clades and species abundances in communities, as we show below.

To infer species motifs, as in previous studies of realms^2–4,17^, we start with an *N* × *G* data matrix of 0s and 1s denoting the absence and presence^18^ of all terrestrial breeding bird species (*g* = 1, 2, …, *G*, here *G* = 9518) resolved to 1° × 1° across the globe (*n* = 1, 2,…, *N*; here *n* = 17,441), respectively. We fix the number of motifs (*K*) a priori and estimate (1) the probability of membership of the *g*th species in the *k*th motif (*θ*_*kg*_) and (2) the proportional contribution of the *k*th motif to the *n*th map cell (*ω*_*nk*_, Supplementary Fig. 1 illustrates the general method). The *ω*_*nk*_ values are displayed using a spatial STRUCTURE plot^19^ where each cell contains a pie chart whose colours indicate the proportional contributions from each motif. We fit the model for many different values of *K* (*K* = 2…35) to compare motifs according to different levels of partitioning. We also computed motifs on the transposed data matrix, where the rows define species and the columns define sites, resulting in a clustering by shared locations^20–22^. Results are reported in Supplementary Fig. 2 and are consistent with those described in the main text.

The methods allow us to bring together data from global biogeography and local community ecology—data that are usually considered separately—into one analytical framework to evaluate the reality of geographically cohesive biotas. At the global scale, we find that transition zones between regions occupied by tropical- or temperate-associated biotas are often narrow and appear to be concomitant with a line of regular freezing, here defined as locations where the mean minimum temperature of the coldest month is < 0 °C. To evaluate consequences at the local level, we show that bird communities in the Himalaya below and above the freezing line are largely populated by different tropical and temperate biotas with affinities to tropical South Asia (India and Southeast Asia) or to temperate Asia (e.g. central and northern China), respectively. We find that the importance of the freezing line is retained when clades rather than species are considered, implying a deep history to these patterns.

## Results

### Global species motifs and the line of regular freezing

**Fig. 1:**
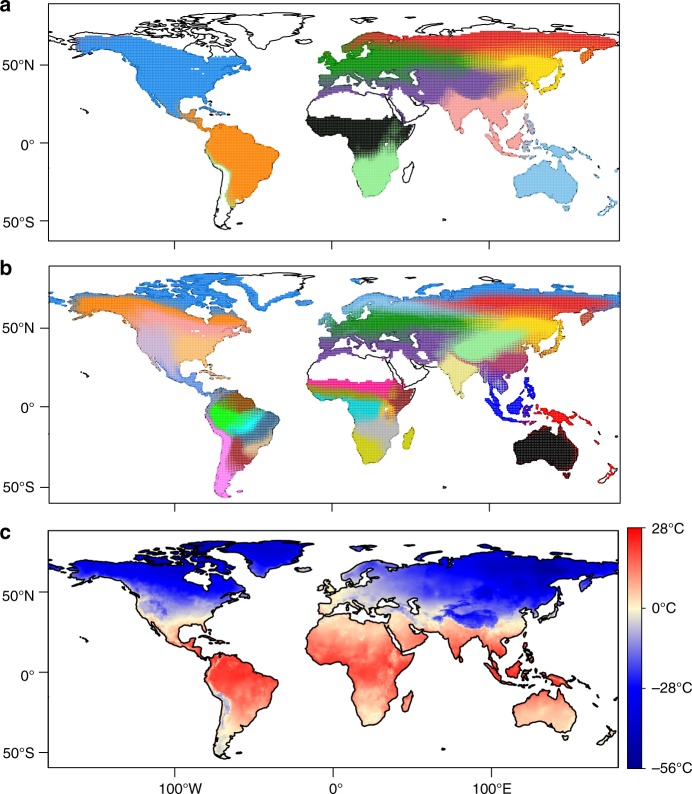
Species motifs for breeding birds. **a** *K* = 11 motifs. Pie charts represent the proportional contribution of species motifs to each 1° × 1° global map cell (i.e. *ω*_*nk*_). Colours represent the different motifs. Blank regions (no pie chart) contain relatively few species and are dominated by a motif that represents locations of low richness. This motif arises because of the dominance of the other ten clusters, which summarize the species composition of the majority of locations across the world. **b** Same as **a** but with *K* = 33. **c** Mean minimum temperature for the coldest month for each map cell (from worldclim.org^43^). The freezing line is indicated by the pale-yellow coloured cells. Note the correspondence between sharp turnover of species motifs in **a**, **b** and the freezing line in **c**. A high-resolution version of this figure, where the pie chart for each map cell can be clearly viewed, is available for download on figshare.com (https://figshare.com/s/2c3f5464cb1190e6602a)

Across the world, most species motifs contribute to geographically cohesive locations, recapitulating zoogeographic regions (Fig. 1). These are generally similar to those identified in earlier studies, but there are differences. For example, even when *K* = 11, sub-Saharan Africa falls into two motifs, bounded approximately by the tropics, whereas Holt et al.^2^ considered this entire region to be 1 of the 20 zoogeographic regions, perhaps because species that span both areas contribute to their metric.

Turnover of motifs within continents often correlates with climatic gradients^5^ (e.g. in precipitation across the southern edge of the Sahara, see black motif in Fig. 1a), but the width of transition zones varies greatly. As can be seen in Central America (orange to blue in Fig. 1a), South America (southern border of orange), and South Asia (pink to purple), a relatively sharp turnover corresponds approximately to the line of regular freezing (compare Fig. 1a, b with Fig. 1c), with possible additional contributions from historical barriers and precipitation^5^. Turnover between tropical- and temperate-associated motifs in mountainous regions is exceptionally steep, similar to that demarcating continental boundaries. The tropical–temperate partition persists across values of *K* from 5 to 35 (e.g. Fig. 1a, b), i.e. arises early in the partitioning and persists through further partitions, which more or less subdivide previously identified realms. The generally steep and persistent turnover across the tropical–temperate boundary contrasts with more continuously varying patterns that arise within the tropics or temperate (e.g. across Central Asia or North America, Fig. 1a, b).

In Asia, the freezing line runs right along the Himalaya (Fig. 1) at ~1500 m (east) to 1700 m (west) (Supplementary Fig. 5e). Ficetola et al.^5^ inferred that the turnover of species across the Himalaya was due to both barriers imposed by the mountains themselves (i.e. orography) and climate. The coincidence of the motif boundary with the freezing line implies a strong role for climate (temperature). Here, locations associated with one motif appear to be separated from locations associated with the other motif by only a few kilometres and no obvious barriers, which makes this the ideal location to assess a realm boundary that is associated with the freezing line.

### Geographical associations of local communities across the Himalayan elevational gradient

**Fig. 2:**
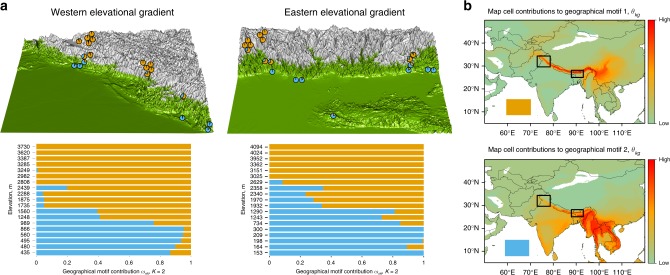
Assignment of regional features to 38 local communities of Himalayan birds, *K* = 2 motifs. **a** Communities arranged by elevation within geographical regions. Colours of each bar represent the proportional contribution of the two geographical motifs to the community’s dispersion field. These same proportions are plotted as pie charts at the locality of each community on three-dimensional maps of elevation in the west and east region; the silver shading represents the freezing line where mean minimum January temperature < 0 °C (Supplementary Fig. 5). The spatial extent of dispersion fields was confined to the area mapped in **b** (*G* = 201,600 map cells). **b** Heat map of proportional contributions of each map cell to each motif (*θ*_*kg*_), with colour intensity relative to the value of *θ*_*kg*_. Rectangles indicate boundaries of regions depicted in **a**

To examine the fine structure of this boundary and how it impacts local communities, we conducted field studies at all elevations across both the east and west Himalaya. We censused bird communities on 38 five ha. sites (Fig. 2a). Unlike the global analysis, we are now restricted to these 38 sites, and yet the questions we address require that we input a broader geographic context. To incorporate geography, we define each community based on its dispersion field, which is the number of species from that community found in each map cell^21,23,24^ (see Supplementary Fig. 3 for a visualization.). Input for this analysis is again an *N* species × *G* map cell matrix (Supplementary Fig. 1 illustrates the method explicitly for this case.). Here, each row represents each community’s dispersion field (*n* = 38 rows), and the cells in the matrix are populated by the numbers of species, rather than 0s and 1s. Hence, we use an underlying multinomial distribution rather than binomial distribution. In this case, clustering depends only on the extent to which communities have similar dispersion fields and is agnostic with respect to species identity. For example, high elevation dispersion fields across the entire Himalaya look very similar (Supplementary Fig. 3) even though associated communities may share only 50% of their species.

We refer to the motifs generated by this analysis as geographical motifs. Geographical motifs are defined by a weighted contribution of map cells (*θ*_*kg*_). The influence of a region on a community is then defined by the proportional contribution of the *k*th geographical motif to the *n*th community’s dispersion field (*ω*_*nk*_). This analysis is robust to the precision of range maps (see “Methods”). Given that the global analysis of species motifs suggests the collision of two motifs along the axis of the Himalayas, we start with an assessment that sets *K* = 2, which also has the highest statistical support (Supplementary Fig. 4 gives model output with values of *K* = 2…5). Proportional contributions of each geographical motif to each community are shown in Fig. 2a, where communities are arranged by elevation along two gradients, one in the east and one in the west. Consistent with the global analysis, turnover is by elevation, not by geography, and occurs between 1200 and 1800 m, i.e. straddling the freezing line (green–grey in Fig. 2a; Supplementary Fig. 5e). This consistency is one line of evidence that motifs are defined by transition between freezing and non-freezing conditions. In particular, with western and eastern sites separated by >1000 km, one might expect geographical distance to partition the system into eastern and western components, but the elevational partition dominates. In Supplementary Fig. 6, we illustrate the underlying causes of the patterns for one group, that of the larger minivets (*Pericrocotus*). In the east, the elevational ranges of three species are largely below the freezing line, and one lies above. The one above and one of those below extend all the way to the west. Largely because of these long thin ranges, measures of beta diversity^25^ for whole communities imply a 5000:1 rule, whereby 1 km in elevation generates as much community turnover as 5000 km in geographical distance (least-squares regressions of phylogenetic distance against elevational distance: *β* = 1.24 × 10^–2^ units/km and geographic distance: *β* = 2.56 × 10^–6^ units/km, Supplementary Fig. 6).

**Fig. 3:**
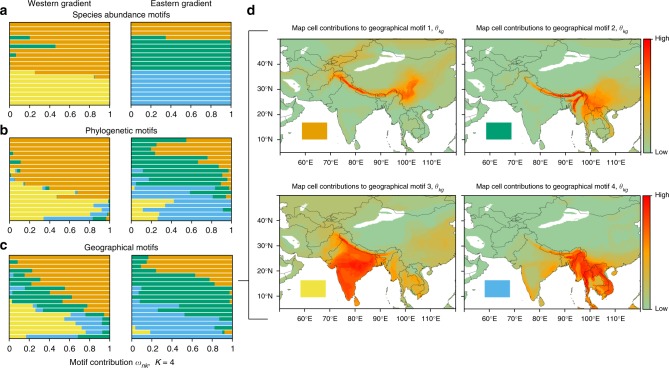
Motif contributions to local communities when *K* = 4. Communities arranged by elevation within geographical regions, as in Fig. 2a. **a** Species abundance motif contributions to local communities. **b** Phylogenetic motifs for clades subtended by the 20 million-year timeline (*n* = 67 clades, Supplementary Fig. 10). **c** Geographical motif contributions to local communities. **d** Maps show map cell contributions to the geographical motifs (*θ*_*kg*_), with colour intensity relative to the value of *θ*_*kg*_

Maps in Fig. 2b show the proportional contribution of each cell to the two geographical motifs. A temperate motif extends out of the Himalaya to the mountains of western China and a tropical motif extends into peninsular India and Southeast Asia. High elevation communities from the west have similar dispersion fields to those from the east (Supplementary Fig. 3). Evidently, a temperate fauna associated with western China tracks climate across the Himalaya, perhaps because no Pleistocene refuge existed to the west^12,26^. At lower elevations, tropically adapted species have colonized from the plains. Especially across the lower elevations, precipitation regimes differ, with India being generally drier than Southeast Asia (Supplementary Fig. 5). The influence of these climatic differences becomes apparent at higher values for *K* (Fig. 3d). Two of the motifs now split low elevations between the west and east. The associations of the motifs with precipitation are shown in Supplementary Fig. 5b–d. The appearance of the west and east motifs may be linked not only to precipitation^12^ but also to mountain barriers^26^, which have been previously correlated with species range extensions from east to west. However, given the primacy of the elevational split at low values of *K* (e.g. *K* = 2), these effects are clearly of lesser importance than controls driven by climatic (temperature) turnover along the elevational gradient, and in particular the transition from tropical- to temperate-associated motifs across the freezing line.

If communities are strongly affected by the climatic regime in which they are embedded, we predict that species from one motif (e.g. tropical) that are present in the other regime (e.g. the temperate) should be at relatively low abundance. To test this prediction, we clustered communities not by their dispersion fields but by the species they contain and those species’ abundances. Here, the columns are all species encountered at least once (*G* = 304 species), the rows are the *n* = 38 communities, and the cell entries are the number of individuals of the species censused in the community. We term the clusters produced species abundance motifs (note that 0 values also contribute to this assessment)*. θ*_*kg*_ is the proportional contribution of the *g*th species to the *k*th motif, and *ω*_*nk*_ represents the proportional contribution of the *k*th motif to the *n*th community’s species abundance distribution. Figure 3a visualizes results for *K* = 4 (see Supplementary Fig. 4 for *K* = 2 to *K* = 5). In all cases, the species abundance motifs (Fig. 3a) are largely congruent with the geographical motifs (Fig. 3c). Most importantly, a transition between distinct species abundance motifs is concomitant with turnover between tropical and temperate climates and is sharp for each value of *K* reported (Supplementary Fig. 4). As a further evaluation of the putative influence of freezing when *K* = 2, we generated competing model fits where we preset the line of partition at varying elevations and compared those fits to the original Grade of Membership model fit. We found strong support for the original model fit using this method (Supplementary Fig. 7). Finally, a post hoc analysis of the *θ*_*kg*_ vectors, which measures species contributions to motifs (Supplementary Data 1) shows that individual species are most often associated with one motif, revealing strong compositional differences between motifs.

When *K* = 4, the top contributing species to each species abundance motif lie along orthogonal axes defined by elevational and east–west partitioning, with many species again uniquely contributing strongly to each motif (Supplementary Data 1). The geographical distribution of these motifs can be visualized in an interactive network of species co-occurrences (Supplementary Fig. 8). Species abundance motifs match those of the geographical motifs with *K* = 4 but show sharper turnover along the elevational gradient (compare Fig. 3a, c). Species abundance motifs also show sharper turnover than they do when species motifs are inferred based on presence/absence irrespective of abundance (Supplementary Fig. 9). These findings support the prediction that species sourced from one region generally have low abundance when they occur in a community sourced primarily from another region.

Results so far suggest a structuring of biological communities according to the climatic regime in which they are embedded (Fig. 2a, Supplementary Figs. 2, 5). If this is to contribute to broad-scale differences between the temperate and the tropical regions, such structuring should have a deep phylogenetic imprint^10,27^. To evaluate this, we generated phylogenetic motifs, continuing to weight by abundance. For each community, we summed the abundances of all species belonging to a given clade and proceeded as in the species abundance motifs. Clades were defined with respect to a given time slice in the phylogeny (Supplementary Fig. 10). Even at the 20 Ma timeline, where the collapsed phylogeny has just *G* = 67 tips, the ensuing phylogenetic motifs show strong structure, which matches that of the geographical and species abundance motifs (Fig. 3; Supplementary Fig. 4). The distribution of phylogenetic motifs along the elevational gradient reflects restriction of whole clades to below or above the freezing line. For example, within the passerines *Cyornis* flycatchers at low elevation are replaced by ecologically similar, higher elevation *Ficedula* flycatchers. Nonpasserines tend to appear below the freezing line^28^, and groups such as the barbets, *Megalaima*, are only found up to the freezing line. Given the persistence of the overall pattern, we conclude that the effects of climate in shaping modern communities have a deep history.

## Discussion

Our analyses of the different motifs reveal a consistent pattern of partition between temperate and tropical climates. Our conclusions draw on the combined assessment of geographical, compositional, and phylogenetic axes of diversity and comparisons of the model fits for different values of *K*. As has been discussed widely in the population genetics literature^29,30^, we do not consider that there is a best value of *K* for any one of these axes nor that values of *K* with lower support are biologically meaningless, especially because significance is tested against an unclear sampling distribution. Rather, additional partitions are of value in assessing the hierarchy of contributions of biotas to realms and communities.

Across many environmental gradients, including within the Himalaya, community taxonomic composition appears to vary more or less continuously, both across elevation and geography (e.g. Supplementary Fig. 6). Based on observations such as these, Ricklefs^31^ argued that continuous turnover implies communities are artificial constructs, whose structure is heavily impacted by dispersal from elsewhere. Results presented here imply a more integrated local community than may be inferred from species lists. First, common species are shared over parts of the gradient, overlapping with common species that are present in other communities, thereby generating a network that can be partitioned into well-supported motifs. For example, most communities contain a common species of sunbird, which occupies a well-defined ecological niche. Most communities also contain one to several small warblers, occupying a different but again well-defined niche. Thus functional similarity of communities extends across the entire gradient. Turnover of species along the elevational gradient is not coincidental across the two groups, but in both cases different species are found above and below the freezing line. Second, if dispersal strongly affects community structure, then communities associated with more than one geographical motif should show elevated species numbers and phylogenetic diversity with respect to communities associated with just one geographical motif. We found no support for this (Supplementary Fig. 11). Species richness varies idiosyncratically both within and between motifs. Phylogenetic diversity monotonically declines with elevation, reflecting the increasing dominance of a single suborder, the oscines (order Passeriformes), which are thought to have become established from 35 million years onwards in association with a cooling climate^28^. Communities with tropical affinities are sourced by older lineages, and this overwhelms any potential effect of mixing on phylogenetic diversity.

In conclusion, by developing methods that allow for continuous variation, we confirm two major insights of Wallace^1^—that the world is indeed organized into discrete zoogeographic regions and that tropical biotas have a long history distinct from that of the temperate. Biological diversity is partitioned hierarchically across the globe, with shallower climatic transitions producing nested and more continuous turnover of zoogeographic regions within the major realms (Fig. 1a, b, Supplementary Figs. 4, 5). The discrete contribution of realms and their boundaries persist even when considering collections of locally interacting species across very steep climatic gradients. This organization may work through conservatism in the direct effects of temperature on bird species ranges and abundances^32^ or through similar conservatism in the distributions of producers, such as trees^10^, which in turn drive consumer coexistence^28^. The deep history and strong geographical restriction of biotas is consistent with explanations for tropical–temperate differences rooted in hypotheses of age, area, and climatic stability^8,10^. The strong linkage of local communities to regions defined by climate confirms that historical influences on community structure and function apply not just between continents but across climatic regimes as well^33^.

## Methods

### The Grade of Membership model

The Grade of Membership model^13^ is a clustering model that allows each sample to have proportional memberships in more than one cluster, where a cluster is defined by the underlying features of interest. Such models are widely recognized in population genetics as ADMIXTURE models^14^, where they are used to determine an individual’s ancestry from multiple populations, with each population having a distinct single-nucleotide polymorphism-level allele frequency distribution. In Natural Language Processing, these models are popularly known as topic models or Latent Dirichlet Allocation models and are used to decompose the semantic structure of text documents into one or more topics, where each topic is defined by a distinct frequency distribution of words in a vocabulary.

Here, we illustrate how the Grade of Membership model can be extended to decompose the structure of surveyed ecological communities into component building blocks of community structure. Valle et al.^16^ previously applied such a model to species abundance data, where they referred to the clusters as component communities. In this paper, we develop a flexible framework that can be used to cluster both taxonomic presence–absence data as well as community abundance data, thereby integrating analyses at the global (i.e. biogeographic) scale with assessments at the community level. At the global level, we assess the structure of species presences to identify patterns of bioregionalization across space. At the local level, we assess structure for various axes of biodiversity—namely species abundances, lineages (phylogenetic), and regions (geographic)—to examine how biogeographic patterns are reflected within ecological communities. We generically refer to the clusters generated by these analyses as motifs. A motif can be viewed as an analogue of ancestral populations in ADMIXTURE models or a topic in topic models.

### Species motifs

In the global context, we constructed a binary presence–absence matrix by overlapping the geographic distributions of breeding birds for each 1° × 1° global map cell meeting two conditions: (1) the cell contained at least 10% land cover and (2) the cell was overlapped by at least three species breeding distributions. The resulting matrix comprised 9518 bird species in 17,441 map cells that passed the processing steps. Polygons of species breeding distributions were obtained from BirdLife International^18^.

We applied a Bernoulli version of the Grade of Membership model on the presence–absence data matrix *M*_*N* × *G*_ *=* ((*m*_*ng*_)), where *m*_*ng*_ is 0/1 based on if the species *g* is absent/present in map cell *n*.

1 $$m_{ng}\sim {\mathrm{Ber}}\left( {p_{ng}} \right)$$

where *p*_*ng*_ is the probability that bird species *g* is present in the map cell *n*. We assume a lower dimensional representation for *p*_*ng*_.

2 $$p_{ng} = \mathop {\sum }\limits_{k = 1}^K \omega _{nk}\theta _{kg}$$

where

3 $$0 \le \omega _{nk} \le 1\quad\quad\mathop {\sum}\limits_{k = 1}^K {\omega _{nk}} = 1\quad\quad\forall n$$

4 $$0 \le \theta _{kg} \le 1\quad\quad\forall k\;\forall g$$

Here, *K* represents the number of underlying motifs fitted in the model, *ω*_*nk*_ represents the proportional contribution of the *k*th motif to map cell *n*, and *θ*_*kg*_ is the probability that the *g*th species is a member of the *k*th motif. We assume non-informative Dirichlet priors on the proportions vector *ω*_*n*._ and non-informative beta priors for each *θ*_*kg*_.

We fit this model for different values of *K* ranging from 2 to 35, and models for different *K* were compared using Bayes factors. The membership proportions vector *ω*_*n*._ for each map cell *n* was displayed using a pie chart, placed at the exact latitude and longitude of map cell *n*. At a 1° × 1° resolution, this visualization shows both the spatial distribution of the motifs and, crucially, the transition between motifs across space (see Fig. 1). The *θ*_*k*._ vector of probabilities represents the probability that a bird species is a member of species motif *k* and can be used to extract the bird species that differentially contribute to the different species motifs.

### Species abundance motifs

Here, the input data comprises community censuses of species abundances in 38 5 ha forest sites across the Himalaya (“Field methods” and “Data availability” described below). We observed 304 species in total and recorded their abundances in a table of counts *C*_*N* × *G*_ = ((*c*_*ng*_)) where *c*_*ng*_ is the number of individuals of species *g* observed in site *n*. Here we applied a multinomial version of the Grade of Membership model that allows each surveyed site to have contributions from multiple motifs, where each motif is defined by the proportional contribution from each species. Each row of the data matrix is modelled as follows.

5 $$\left( {c_{n1},c_{n2},\cdot \cdot \cdot ,c_{nG}} \right)\sim {\mathrm{Mult}}\left( {c_{n + },p_{n1},p_{n2},\cdot \cdot \cdot ,p_{nG}} \right)$$

where $c_{n + } = \mathop {\sum}\nolimits_{g = 1}^G {c_{ng}} \;{\mathrm{and}}\;\mathop {\sum}\nolimits_{g = 1}^G {p_{ng}} = 1$ for all *n*. As in case of species motif model, we assume that *p*_*ng*_ has a low rank structure and can be decomposed as

6 $$p_{ng} = \mathop {\sum }\limits_{k = 1}^K \omega _{nk}\theta _{kg}$$

where

7 $$0 \le \omega _{nk} \le 1\quad\quad\mathop {\sum}\limits_{k = 1}^K {\omega _{nk}} = 1\quad\quad\forall n$$

8 $$0 \le \theta _{kg} \le 1\quad\quad\mathop {\sum }\limits_{g = 1}^G \theta _{kg} = 1\quad\quad\forall k$$

Here, *K* represents the number of motifs, *ω*_*nk*_ represents the membership proportion of the *k*th motif in site *n*, and *θ*_*kg*_ is the relative frequency of the *g*th species in the *k*th community. We assume non-informative Dirichlet priors on the membership proportion vector *ω*_*n*._ for sample *n* and the proportional motif contribution vector *θ*_*k*._ for the cluster *k*.

We fit the Grade of Membership model for different values of *K* from 2 to 10. For each value of *k*, the model was run 10,000 times with different starting points from which the fit with the highest Bayes factor was selected as the best model for that value of *K*. We used the maptpx package^34^ in R to fit the model for each *K*.

We displayed the membership proportions vector *ω*_*n*._ for each community *n* using a Block Structure plot, a slightly modified version of the Structure plot representation used in population genetics for visualizing admixed individuals^19^. In the Block Structure plot, the vectors of membership proportions are represented as horizontal stacked bar charts with samples from the eastern and western Himalaya displayed in parallel blocks and samples for each block ordered by site elevation (elevations are given in Fig. 2 and Supplementary Table 1). This aids in identifying the motif transition points with respect to elevation and comparing the motif structure between regions. The *θ*_*k*._ vector provides the normalized abundance profiles of the different species in motif *k* and can be used to determine bird species that contribute most heavily to the local species abundance motifs.

In this case, species are considered as independent entities, but in reality, they may be related phylogenetically or in terms of morphological characteristics or geographic range distribution. To capture these characteristics, we consider data with the same surveyed sites along the rows but with the features along the columns placed in appropriate units for different axes of diversity.

### Phylogenetic motifs

We obtained a phylogenetic tree of species in our surveyed sites^28^ and cut the tree at a certain time slice, *T*, at which point some lineages in the tree would subtend one or more related species (Supplementary Fig. 10). We obtained a count per site for each tip present in the phylogeny at time slice *T* by summing over the counts of the species that tip subtends. We then fit the multinomial Grade of Membership model (see “Species abundance motifs” above) to the count data matrix with 38 sites along the rows and the common ancestors to species at time *T* along the columns. We considered values of *T* varying from the present day (the tips of the phylogenetic tree) to 20 million years before present. Finding consistent patterns for each 5 million-year increment from the present back to 20 million years before present, results presented in the main text (Fig. 3b) correspond to an analysis with *T* = 20 million years before present. Daru et al.^17^ develop similar methods for incorporating phylogenies in evaluating presence–absence data. Here, we apply this approach to community survey data and model the output with the Grade of Membership model.

### Geographical motifs

For the geographical case, we used rasters, one for each surveyed 5 ha site, to represent the geographic distributions of the species present in each community. We counted the number of species from the community that are present in each raster cell in the region represented in Supplementary Fig. 3 (longitudinal and latitudinal limits, 50°E, 120°E, 5°N, 50°N). This raster is commonly referred to as an assemblage dispersion field^23^, but here we focus on the dispersion field for breeding birds recorded in a small 5-ha area, rather than all birds recorded in the entire raster cell as used in other applications. Polygons of species breeding distributions were provided by BirdLife International^18^. After overlaying the species geographic distributions on a 360-by-560 raster (1/8th square degree cells), we obtained a count per raster cell, corresponding to the number of species with breeding polygons overlapping that cell. We subsequently vectorized the raster and stacked these vectors together to form a 38-by-201,600 matrix of counts, to which we then applied the multinomial Grade of Membership model. Note that these distribution maps are coarse grained and it has been suggested that they cannot be used at resolutions < 110 km^2^ (1° × 1°)^35,36^. However, this is not an issue in our case, where we only need to robustly capture the general features of the dispersion fields in a limited number of motifs and each species list is known from a site-specific survey.

### Modelling considerations

For the Himalayan data, we compared Grade of Membership models for different choices of *K* ranging from 2 to 10. As noted above, to account for the random initialization biases, for each *K*, we ran 10,000 iterations for each of the phylogenetic and taxonomic counts data and 100 for the geographical counts data, and we chose the model fit with the highest Bayes factor for that value of *K*. Because of computational constraints, we ran one iteration for each value of *K* for the global analysis. The results are coherent across different values of *K* (e.g. patterns are often nested for higher values of *K*), implying that the revealed structure is robust.

The Dirichlet prior assumption in the Grade of Membership model assumes that the features along the columns of the data matrix are only weakly dependent. This assumption is not true for the species or species abundance motif studies, as it ignores the phylogenetic relatedness and morphological similarity of species. The phylogenetic analysis captures this relatedness to some extent, but a more comprehensive model that accounts for all inherent relatedness would be more optimal. Also, in the geographic context, the model ignores the spatial proximity of the raster cells to one another. The models we propose thus have room for improvement, but the simplicity of the models make them computationally fast, and as is evident from Figs. 1–3, the approach is successful in decomposing biodiversity of the global map cells or the local Himalayan sites into biologically interpretable motifs. Because of the simplicity of the model, Bayes factors used to compare model fits should be interpreted with caution. General patterns that are consistent across different values of *K* are likely more reliable to interpret than patterns associated with one best fit model.

### Model comparisons

A longstanding concern in community ecology is whether the data conform to an appropriate null expectation. To address this issue, we randomly permuted the Himalayan census data according to four different null model approaches—frequency, richness, independent swap, and trial swap^37,38^. These permutations were generated using the R package picante^39^. We then fit the multinomial Grade of Membership model on the permuted data set and compared, for the same values of *K*, the observed and the null model generated distributions using Bayes factors. For each approach, the null model Bayes factor distribution came from fitting Grade of Membership model for a fixed number of clusters *K* on 1000 null model matrices. We compared the Bayes factor from fitting Grade of Membership models for same number of clusters *K* on the observed census data with respect to the null model Bayes factor distribution. The *p* values were significant with respect to 5% level of significance in all comparisons (Mann–Whitney *U* test, *p* < 0.01).

### Comparison to other clustering methods

The Grade of Membership model uses a low-dimensional factorization to cluster the samples, which calls for a comparative analysis of our approach with other clustering algorithms and dimension reduction methods. In the context of RNA-sequencing data, Dey et al.^40^ showed that the multinomial Grade of Membership model is more efficient in distinguishing samples from known groupings than hierarchical clustering. A possible explanation for this is that the multinomial model is able to capture sparse count data better than a non-model based hierarchical clustering approach, as exemplified by RNA-seq. Sparse count-based data are typical characteristics of species abundance data too, with our data having ~90% sparsity; hence the multinomial Grade of Membership model would seem like a natural choice over hierarchical clustering in this case.

For further evaluation, we compared the taxonomic Grade of Membership results to patterns obtained using two traditional ordination techniques—principal components analysis (PCA) and non-metric multi-dimensional scaling (NMDS). We also compared these results against patterns obtained using t-distributed stochastic neighbor embedding (t-SNE), a more recently developed machine learning method for low-dimensional data projection^41^ (see Supplementary Fig. 12). Since t-SNE is sensitive to the choice of the perplexity parameter^42^, we performed additional visualizations of the taxonomic data using t-SNE at various levels of perplexity. Results were consistently less informative. Unlike PCA, NMDS, or t-SNE, the Block Structure plot representation of the Grade of Membership model displays site membership patterns for multiple motifs in one plot and also highlights change in these motif patterns across elevation and geography much more effectively than other approaches.

### Field methods

Details on the bird surveys are in Price et al.^28^, who present data and describe the methods for 18 east Himalayan 5 ha sites. For this study, in order to account for geographical variation between the east and west Himalaya, T.D.P. and/or D.M. surveyed one additional site in the northeast Indian hills of Meghalaya and 19 sites in the west using similar methods (Fig. 2a). Some sites were surveyed in multiple years. For each site, we use results from a single observer from a single year, but results are not affected if we use other years or switch observers for sites which both T.D.P. and D.M. surveyed (which were the majority). We measured abundance of species belonging to 11 orders (excluding game birds and birds of prey)^28^. In total, we encountered 304 species out of 621 species in these orders that breed in the Himalaya^28^.

### Reporting summary

Further information on research design is available in the Nature Research Reporting Summary linked to this article.

## Supplementary information

Supplementary Information

Reporting Summary

Description of Additional Supplementary Files

Supplementary Data 1

Supplementary Data 2

## Data Availability

Local community bird surveys (species abundance data) and the phylogeny of all birds included in the study are all publicly available in the R package ecostructure. Species abundances in Himalayan communities are also provided in Supplementary Data 2. Species range polygons are available upon request from BirdLife International (http://datazone.birdlife.org/species/requestdis).

## Code availability

Code for fitting Grade of Membership models, analyzing motifs, and generating visualizations is publicly available in the new R package ecostructure. The package contains: (1) functions for processing local community data using a phylogeny to fit phylogenetic motifs, (2) functions for generating presence–absence matrices and dispersion fields from GIS data sources to fit species and geographical motifs, and (3) functions for visualizing the output of those model fits. Tools for null model comparisons and comparisons of geographical motifs with climatic data are also provided. The package, and a detailed vignette of its functionality, is available for download at https://kkdey.github.io/ecostructure.
