# Supplementary material for: Regional influences on community structure across the tropical-temperate divide
Source: Nat Commun. 2019 Jun 14;10:2646. doi: 10.1038/s41467-019-10253-6 (PMC6570764; doi:10.1038/s41467-019-10253-6)
Supplement: Supplementary file 1 — Supplementary Information [file 41467_2019_10253_MOESM1_ESM.pdf]

Regional influences on community structure across the tropical-temperate divide

White et al.

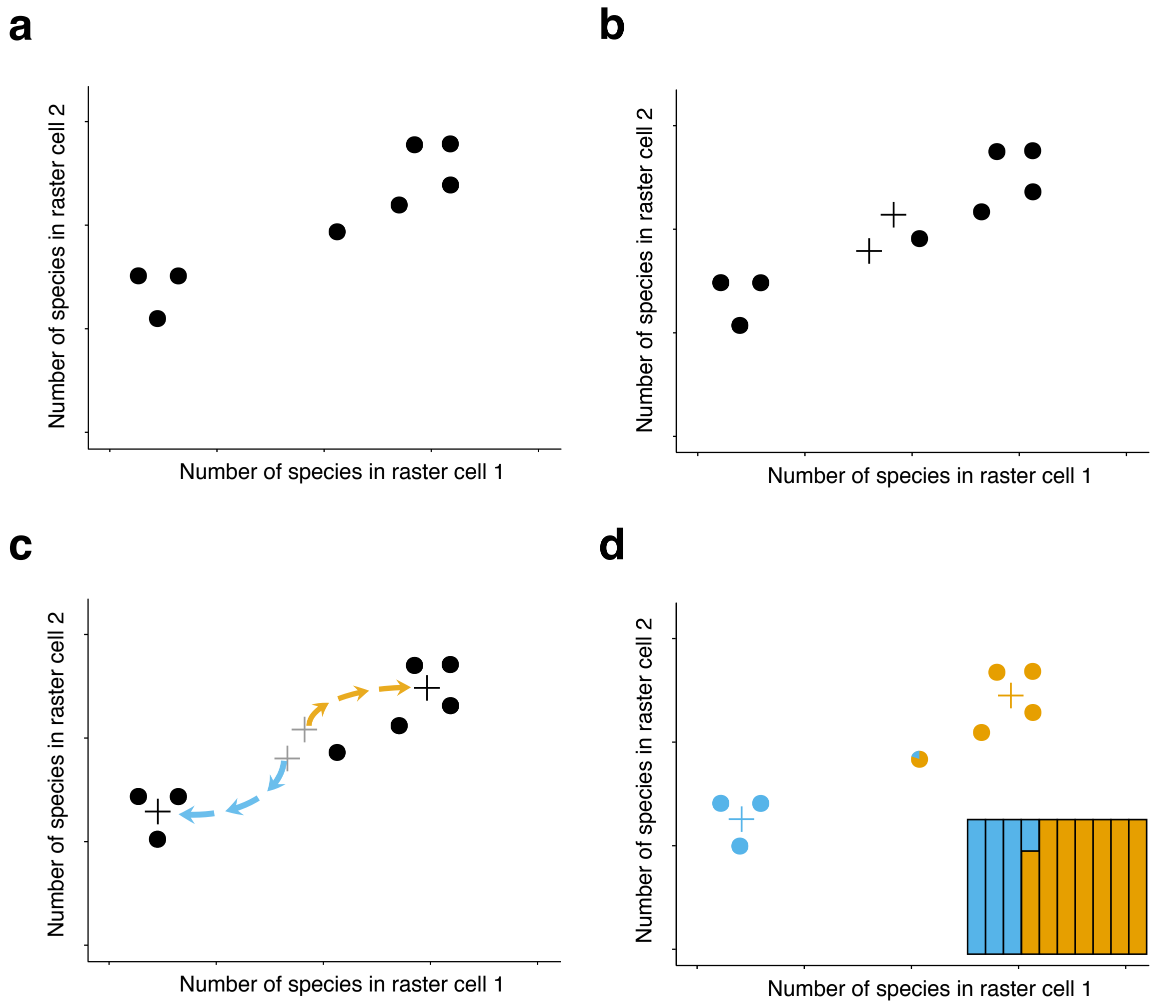

Supplementary Figure 1 | Graphical illustration of the method used to assign species in a localized area or community to geographical motifs. a, The points represent the number of species from each of 8 hypothetical dispersion fields (associated with 8 localities) that are drawn from two cells in a hypothetical raster (note, in our analysis there are 201,600 cells). **b,** The procedure starts with a specific number of motifs, $K = 2$ in the illustrated case. The points are randomly assigned to a motif with equal probability, and their values averaged across the localities assigned to that motif. By chance, the motif means, illustrated by crosses, tend to each lie closer to one of the natural clusters in the data than the other. **c,** In an iterative procedure, localities are again assigned to motifs, but with a probability that is positively correlated with proximity to each. The new average values move towards natural clusters. **d,** The procedure reaches an equilibrium, at which point relative probabilities of a community being assigned to a given motif are recorded (vertical bars, ω_{nk}). Contributions of map cells to motifs (θ_{kg}) can be similarly assigned, e.g. map cell 2 more strongly contributes to the blue cluster than map cell 1 (e.g. see Fig. 2b, Fig. 3). This basic process can be generalized to evaluate other types motifs explained in this paper.

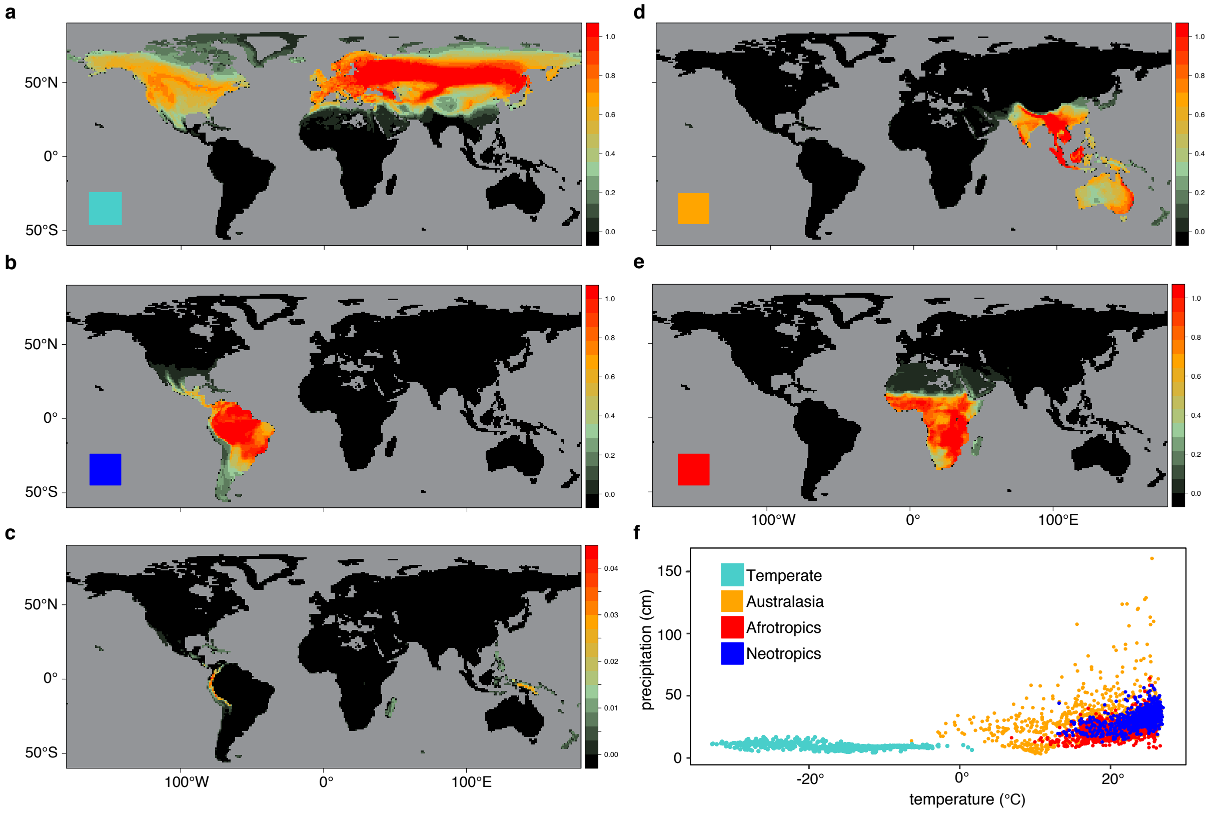

Supplementary Figure 2 | Range motifs. Map cell contributions to motifs defined by species ranges. Here we use a transposed version of the grid × species matrix (on the same data matrix as Fig. 1), where entries are 0 or 1, signifying absence or presence of the species in the cell. The method now clusters map cells, not species, and the clustering is according to shared species ranges; hence we term them *range motifs.* The Grade of Membership model is here fit for $K = 5$. **a-e,** Maps show the probability of membership for each map cell to the given motif (the θ_{kg} from these model fits). Map in the lower left (**c**) represents a motif that captures regions of endemism, a novel feature of this approach. The motif arises because of the dominance of the four other clusters, which summarize the majority of species distributions across the world. **f,** Mean temperature of the coldest 3 months of the year (°C) against precipitation of the wettest month (cm, from worldclim.org[1]) for the top 5% of locations contributing to the 4 major motifs.

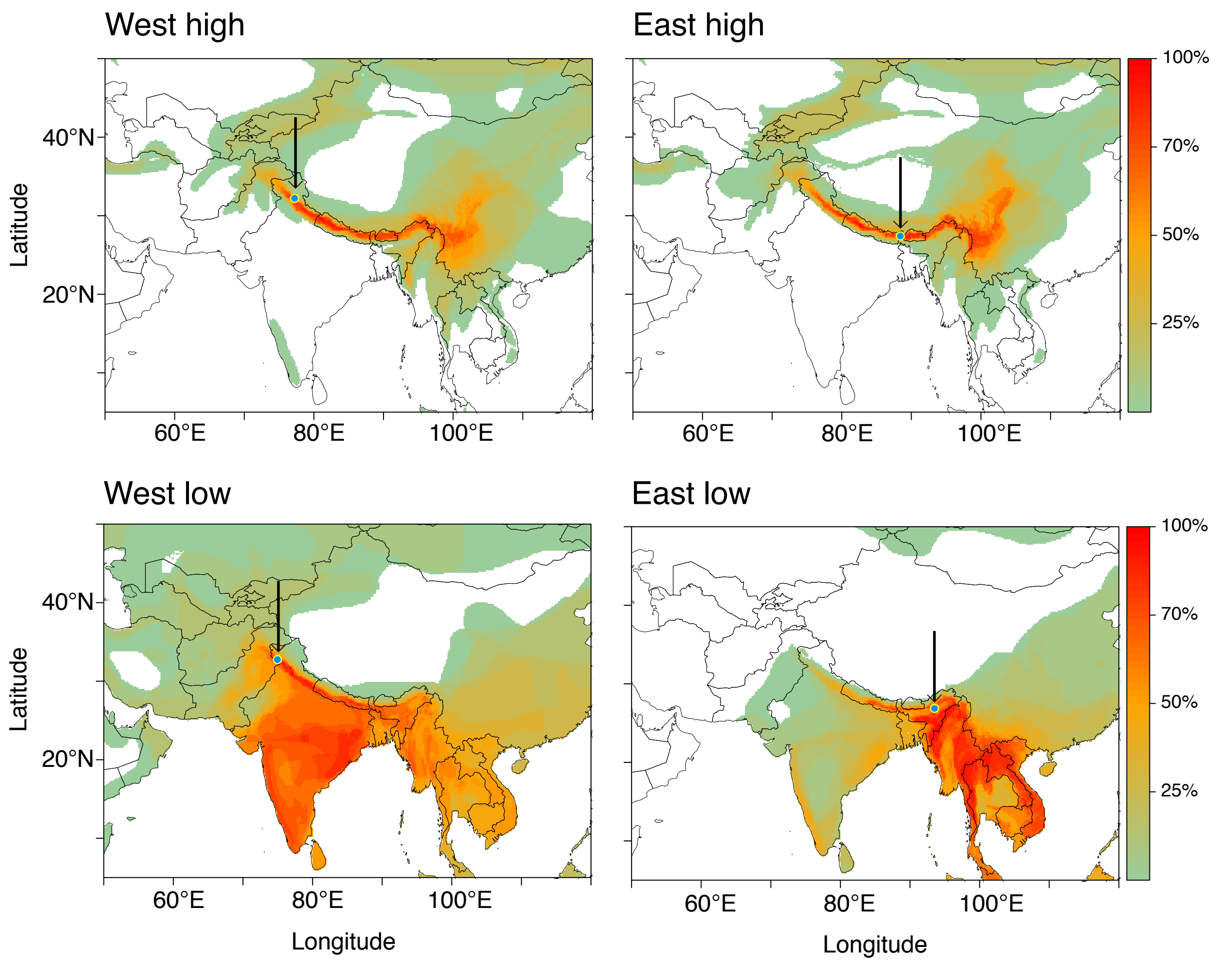

Supplementary Figure 3 | Dispersion fields for four communities. West high (Site: M2, N = 26 species, elevation = 3285 m; see Supplementary Table 1), East high (N2, 20 species, 3952 m), West low (J1, 27 species, 480 m), East low (A8, 28 species, 300 m). Colours indicate the proportion of species from the local site that are present in each raster cell. Locations of each site are indicated by an arrow.

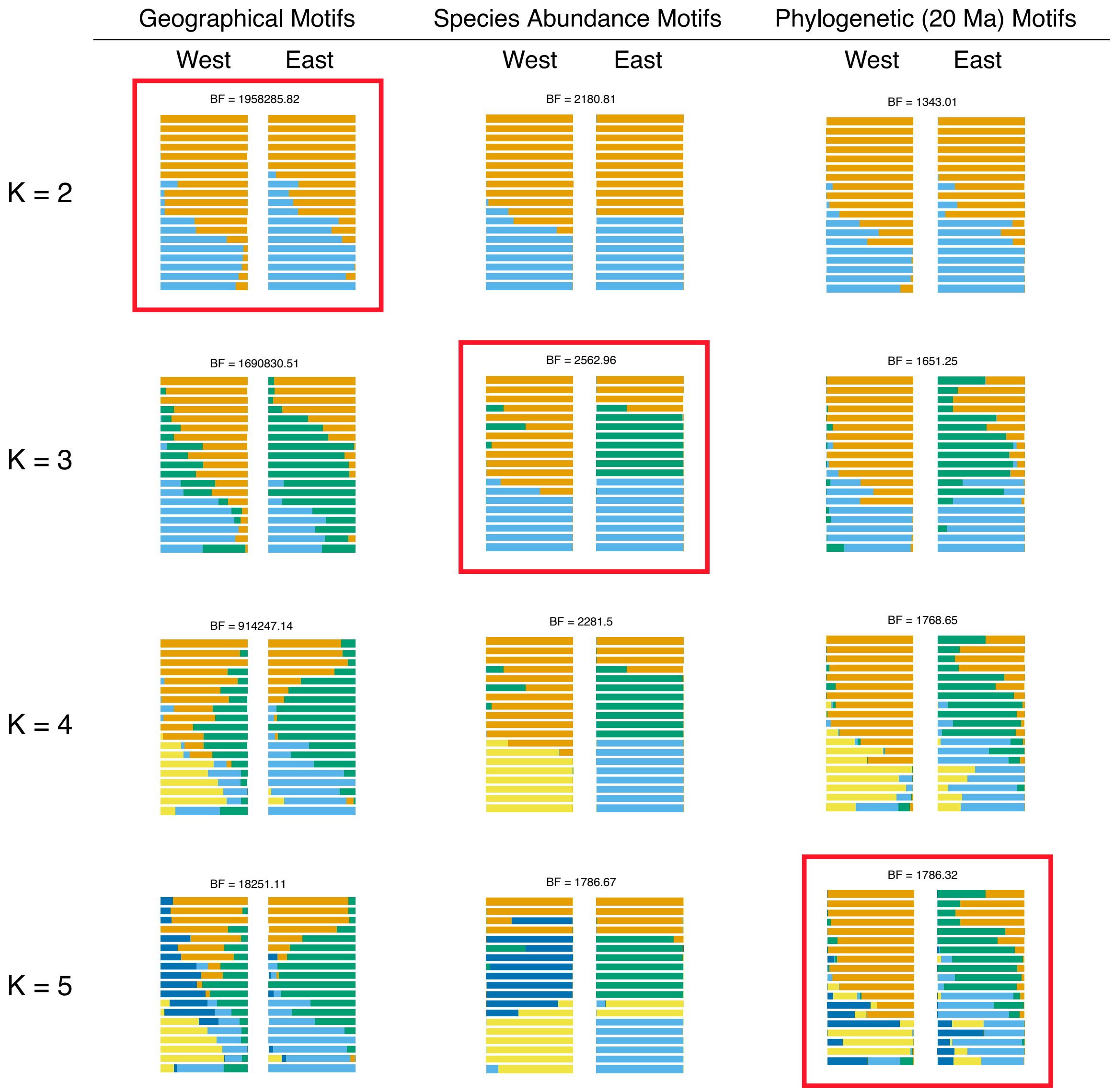

Supplementary Figure 4 | Motifs for *K* = 2-5. Motif contributions to Himalayan bird communities for geographical, species abundance, and phylogenetic (clades subtended at 20 million years) motifs, with associated Bayes factors above each plot. For each column, the *K* value with the highest Bayes factor is highlighted with a red box. Communities are separated into west and east and ordered by elevation within a region.

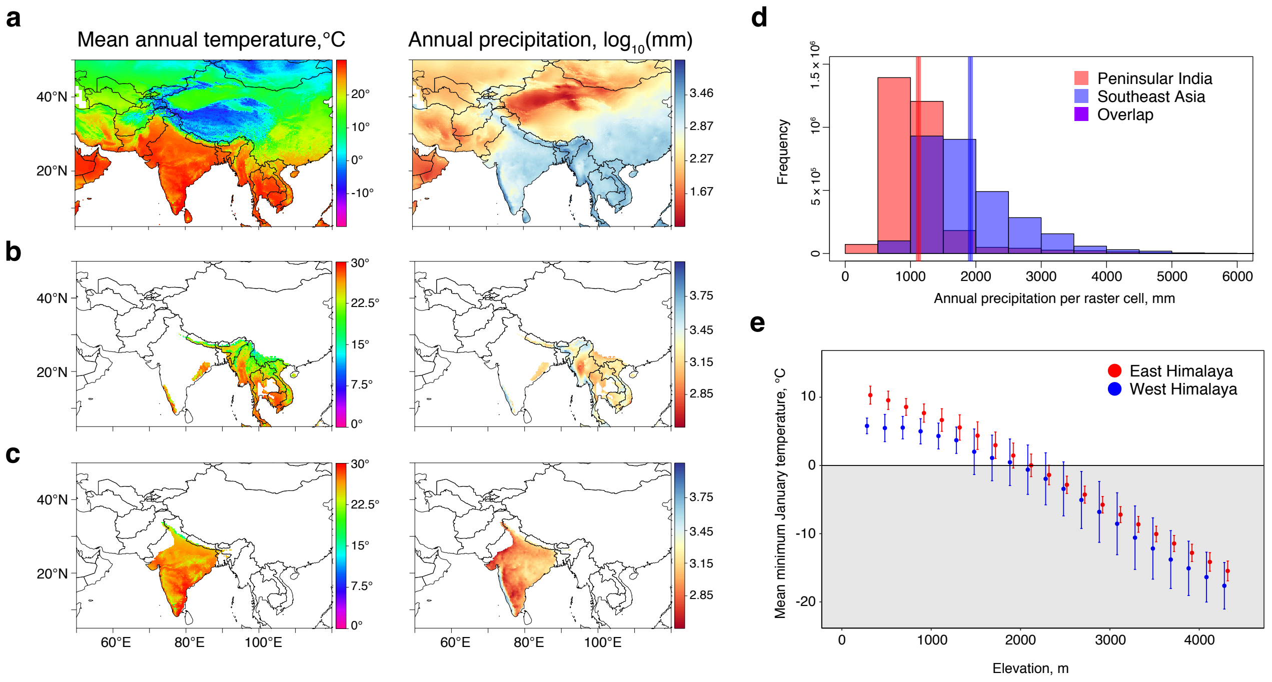

Supplementary Figure 5 | Climate across the Himalaya. a, Mean annual temperature and total precipitation (from worldclim.org[1]). **b, c,** The same climate variables for the top 10% of raster cells contributing to the low-west and low-east regional motifs respectively, when $K = 4$ (see Fig. 3 in the text). **d,** Histogram of the (**b**) and (**c**) cell entries for precipitation showing the east regional motif receives more rainfall. Lines represent mean precipitation in each region. **e,** Mean minimum January temperature against elevation for the west and east Himalayan regions (boundaries for climate were defined by the rectangles in Fig. 2b). Grey shading represents regular freezing.

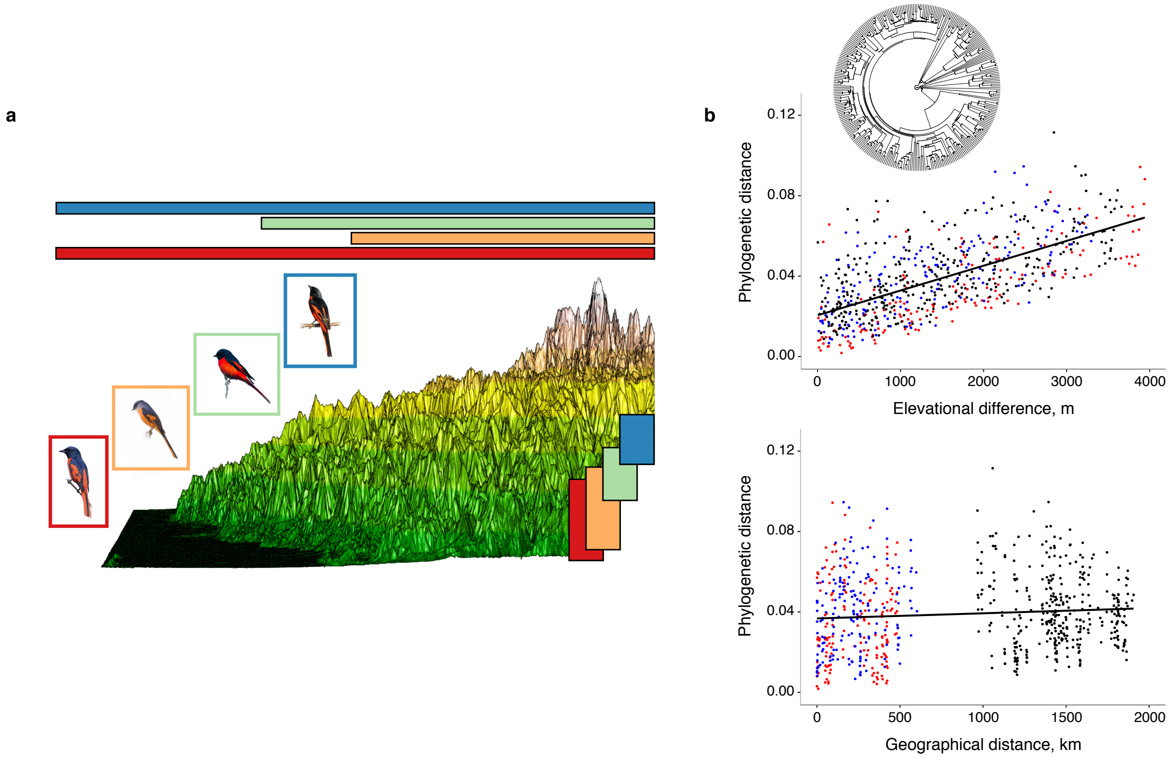

Supplementary Figure 6 | Elevational and geographic similarities of communities. **a,** Horizontal bars at top indicate geographical extent of four species of minivets, *Pericrocotus,* a genus containing species across the tropical-temperate divide. Illustrated from left: *P. speciosus* (red, 150 – 2000 m in the east Himalaya, photo © Raj Phukan), *P. solaris* (orange, 600 – 1900 m, photo © Arthur Grosset), *P. brevirostris* (light green, 1700 – 2800 m, photo © Francesco Veronesi), and *P. ethologus* (blue, 2400 – 3600 m, photo © Myron Tay). Vertical bars at bottom left indicate elevational distributions of the four species in the east Himalaya **b,** Phylogenetic beta diversity, a metric based on phylogenetic distance between species in two communities, weighted by species abundances (phylogeny from ref. 2 is illustrated). Methods for assessing pairwise similarities or distances between communities have proliferated[3]. We use a similarity metric which is inversely proportional to the average within site species phylogenetic distances relative to the average between site species phylogenetic distances, weighted by species abundance (S_Q calculated using $S_{Sørensen}$)[4]. Red points compare sites in the east Himalaya, blue in the west Himalaya and black between the east and west Himalaya. Lines are least squares regression lines fit to all the points (elevation: $\beta = 1.24 \times 10^{-2}$ units/km geography: $\beta = 2.56 \times 10^{-6}$ units/km). Communities separated by 500 m. of elevation differ as much as they do from one end of the Himalaya to the other (~2,000 km), partly because many species have long, elevationally-restricted ranges, as illustrated by the minivets in panel **a**.

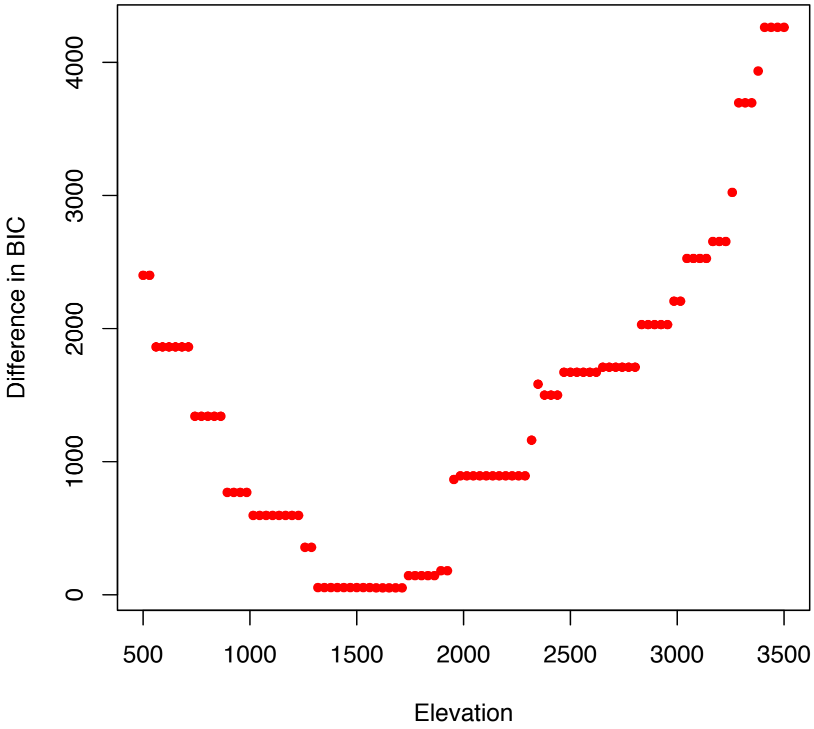

Supplementary Figure 7 | Modeling a single partition (*K* = 2) across elevations. Competing models were generated by setting cluster memberships a priori above or below a particular elevation for *K* = 2 using the species abundance dataset. The difference in BIC is plotted for each preset elevational partition compared to the original model fit using the Grade of Membership model with *K* = 2. The strength of evidence strongly implicates a partition between 1500-1800 m, where the freezing boundary spans the Himalaya between the east and west (Supplementary Fig. 5e).

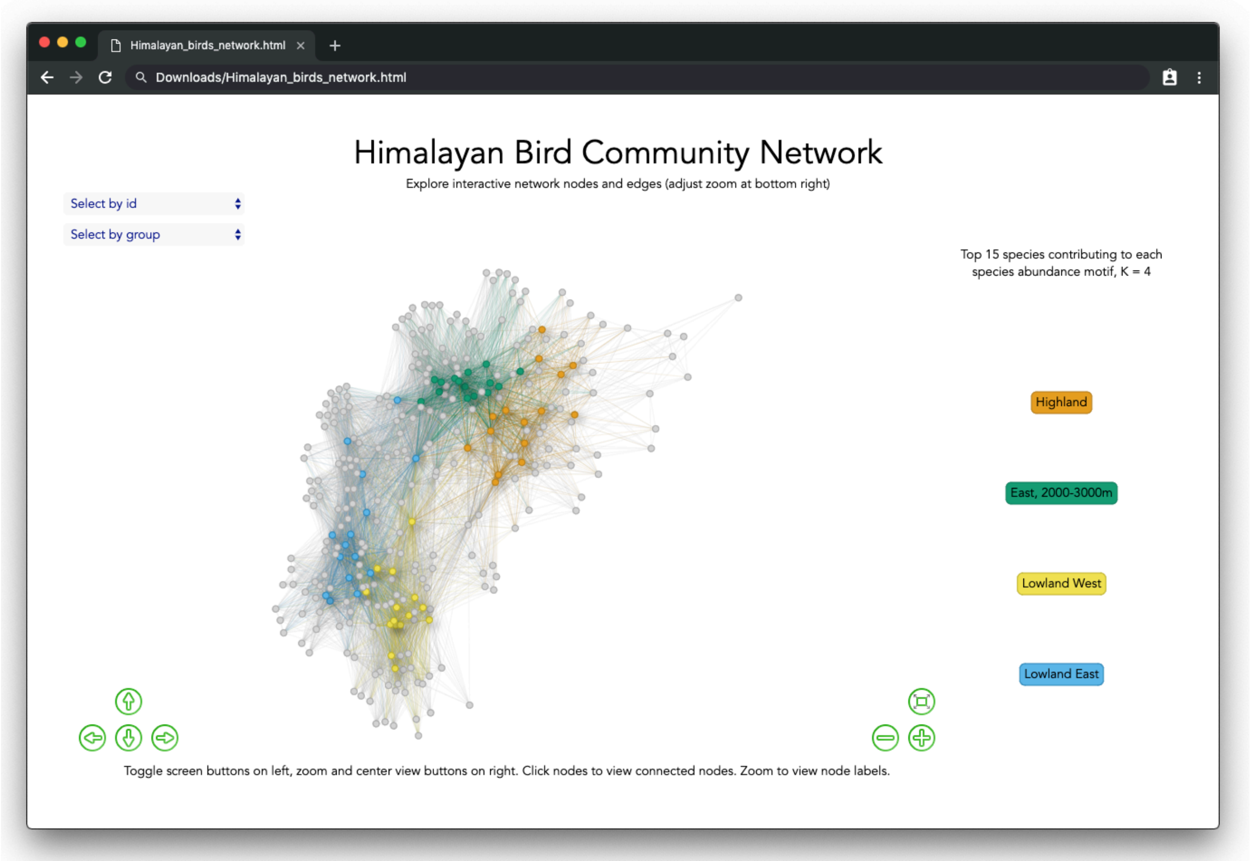

Supplementary Figure 8 | Screen-shot of interactive network of species co-occurrences. A fully interactive version to be opened in an internet browser highlights the species names and their associations (download at https://figshare.com/s/8f605825d4956ac20f0f). Coloured points represent the top 15 species contributing to each of the species abundance motifs (i.e. largest θ_{kg}), when $K = 4$ (Fig. 3a). In the few cases when a species was shared between motifs in the top 15, the species is illustrated with the motif for which it contributes with a higher θ_{kg} value. The next species down the list was then included for the other motif. Edges represent a co-occurrence between species in a community. If two species tend to co-occur or co-occur with similar species they are drawn closer together in the network. Note that species abundance motifs (i.e., colours in the network) are generated using abundances, yet the network structure represents species presences.

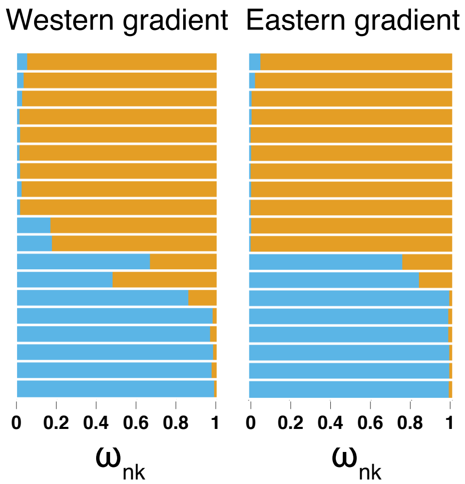

Supplementary Figure 9 | Species motifs generated using a presence-absence model for *K* = 2. These motifs are constructed in a similar way to the species abundance motifs (Supplementary Fig. 4), but species abundances are set to 0 or 1 (i.e., a binomial distribution underlies the Grade of Membership model). Clustering by elevation is retained for $K = 2$, but the sharp transition between tropical and temperate taxonomic motifs found using the multinomial model is more gradual, indicating that abundance distributions sharpen the partitioning associated with species presences in a community.

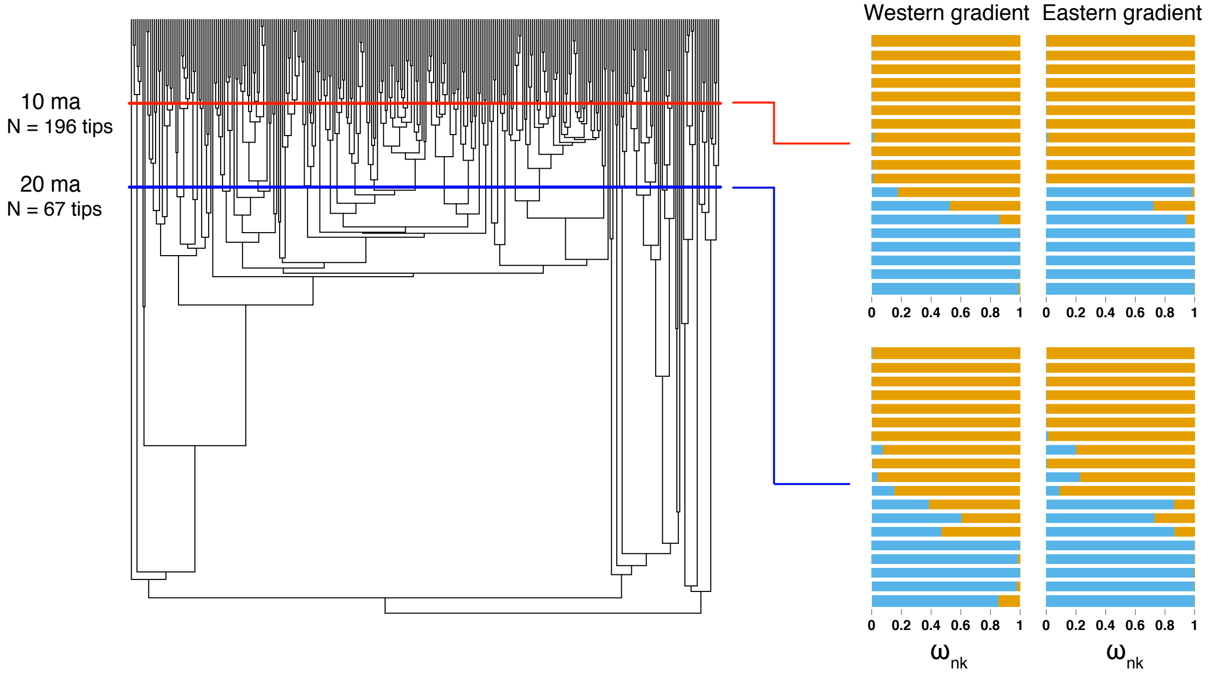

Supplementary Figure 10 | Phylogenetic motifs at the 10 Ma and 20 Ma timelines. These motifs are constructed in a similar way to the species abundance motifs, but with all species in a clade subtended at a timeline collapsed into one lineage, whose abundance in the community is given by the summed abundances of all species in the clade. Strong clustering by elevation is retained for $K = 2$, implying that at least in some clades, the majority of species contribute mostly to one motif.

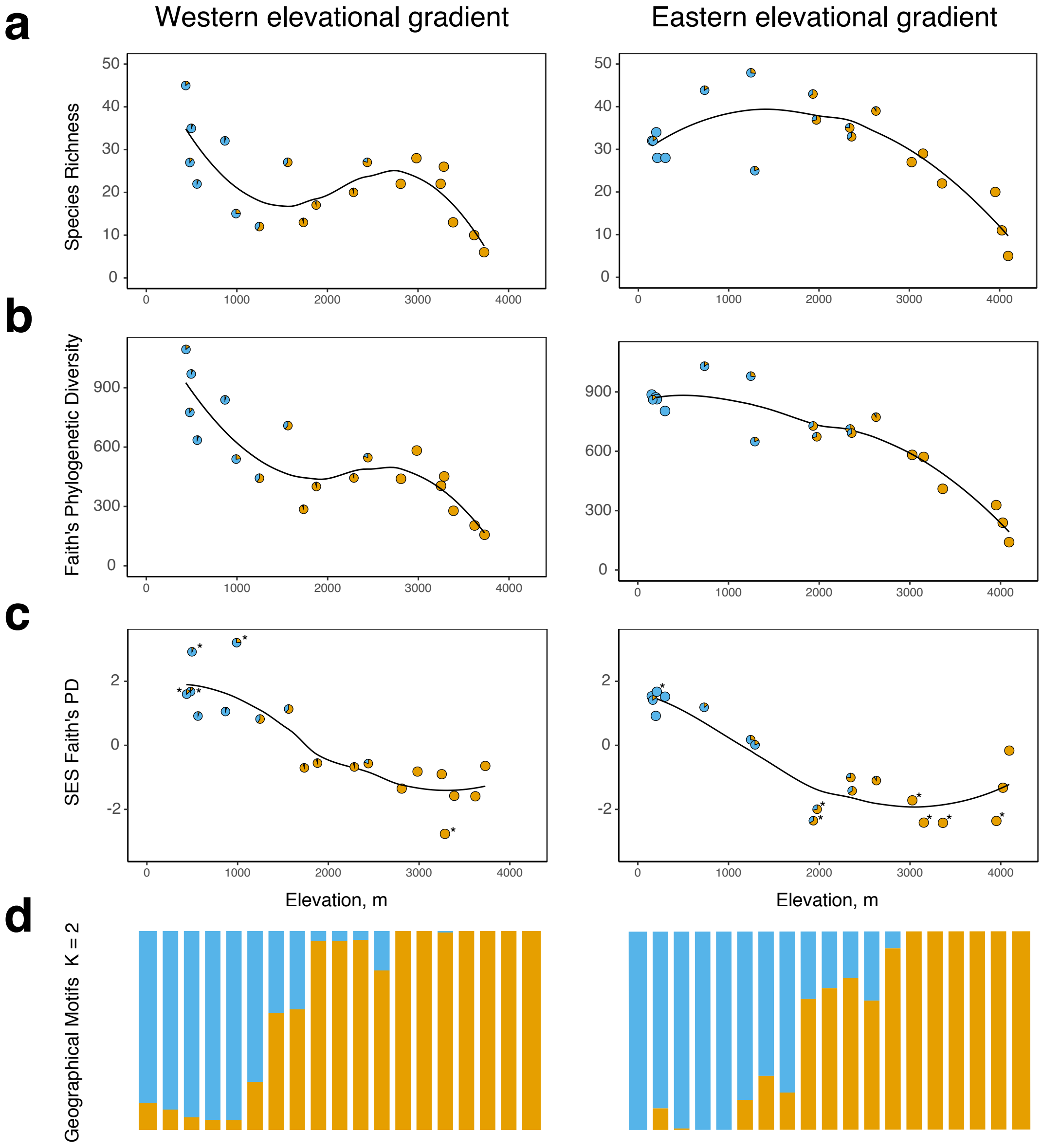

Supplementary Figure 11 | Species richness in communities and phylogenetic alpha diversity in communities. a, Species richness plotted against the elevation of each community, arranged by region. **b,** Faith's phylogenetic diversity (PD, abundance weighted)[4] plotted against elevation. **c,** Standardized effect size of Faith's PD vs. elevation. PD measures the total branch length contained in a phylogeny that connects all species in the sample. Asterisks (*) designate significantly dispersed or clustered communities compared to 1000 simulated communities of the same richness. **d,** Regional motifs with sites ordered along the elevation axis in each region (*K* = 2). All points are colour coded according to the proportional contribution of regional motifs when *K* = 2. Note that regional mixing does not elevate richness or phylogenetic diversity.

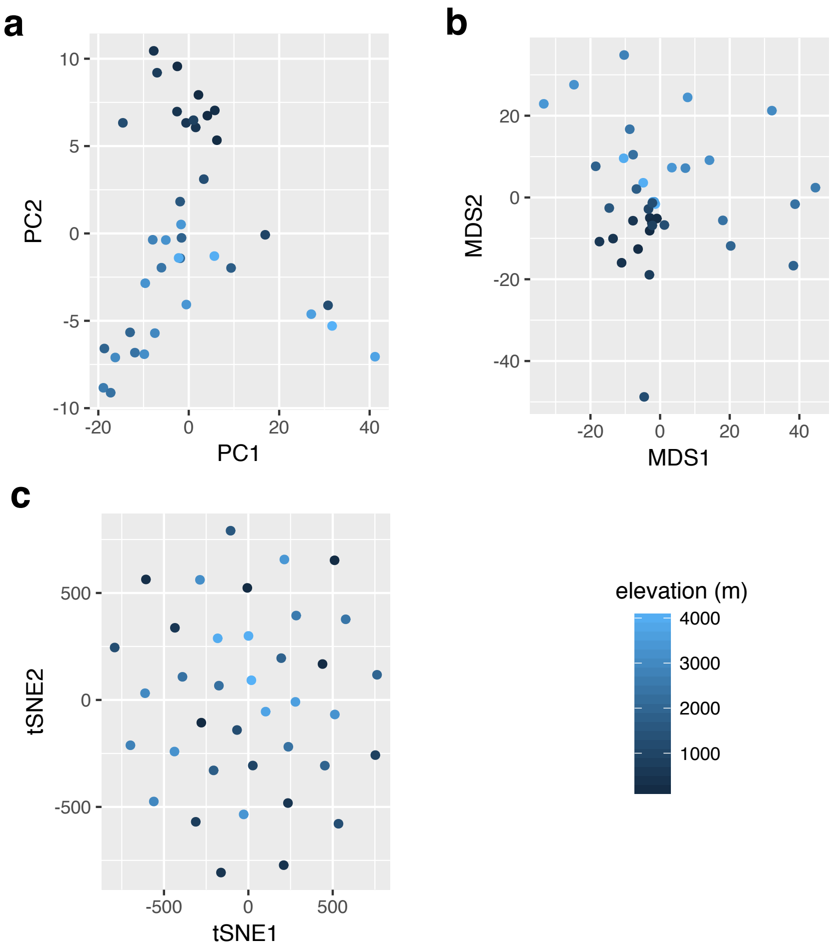

Supplementary Figure 12 | Comparisons with other methods to project similarities of local Himalayan communities. Each panel represents different projections of the local Himalayan site by species census data - **a,** Principal components analysis, **b,** Non-metric multidimensional scaling, and **c,** t-SNE. Each community is coloured according to its elevation. Note that the clustering of sites and patterns of turnover that are revealed when fitting the Grade of Membership model are difficult to interpret when using these approaches.

Supplementary Table 1 | Site metadata for locations of Himalayan bird community surveys.

Site	Elevation (m)	Latitude	Longitude	Region
**B3**	153.5	26.63125	89.5832417	E
**B2**	164.3	26.6293833	89.5829583	E
**A2**	198.3	26.9789833	92.921975	E
**B4**	209.8	26.8964225	88.8559325	E
**A8**	300	26.96138	93.01216	E
**A3**	734.3	27.0062667	92.404575	E
**A4**	1243.3	27.0274958	92.4104083	E
**MA**	1290	25.441827	90.437366	E
**B1**	1932.3	27.061125	88.7706667	E
**B5**	1970.3	27.063185	88.77039	E
**A7**	2340.3	27.0919792	92.4085667	E
**S1**	2358.8	27.416375	88.1979417	E
**A6**	2629	27.1477333	92.4593792	E
**N1**	3025.8	27.7701917	88.5212333	E
**G1**	3151.5	27.4454667	88.1752917	E
**N3**	3362.3	27.8222792	88.5526042	E
**N2**	3952.3	27.8949083	88.5281125	E
**D1**	4024.3	27.469675	88.1646708	E
**D3**	4094.3	27.4773417	88.1626167	E
**U1**	435	29.721111	78.5863889	W
**J1**	480.5	32.7551542	74.8668208	W
**U3**	495.8	30.1634125	77.9147375	W
**J6**	560.3	32.7658525	74.870395	W
**U2**	866.5	29.7915278	78.6261111	W
**J5**	989.3	32.9797325	75.12148	W
**U4**	1248	31.0145542	78.0477375	W
**J4**	1560.8	33.10009	75.12887	W
**K5**	1735	34.14562	74.9190225	W
**J2**	1875	33.0710792	75.330475	W
**K6**	2288.3	33.950585	75.251605	W
**M3**	2439.8	32.2467292	77.1611583	W
**K2**	2808.5	33.975725	75.2413875	W
**M1**	2982.7	32.2416458	77.1310333	W
**K1**	3249.8	33.9784042	75.249475	W
**M2**	3285	32.2360583	77.1272125	W
**L1**	3387.5	32.5005333	76.9817042	W
**K4**	3620.3	33.9867208	75.2522417	W
**M4**	3730	32.2275542	77.1324167	W

Supplementary References

1. Hijmans, R. J., Cameron, S. E., Parra, J. L., Jones, P. G. & Jarvis, A. Very high resolution interpolated climate surfaces for global land areas. *Int. J. Climatol.* **25,** 1965–1978 (2005).
2. Price, T. D., Hooper, D. M., Buchanan, C. D., Johansson, U. S., Tietze, D. T., Alström, P., Olsson, U., Ghosh-Harihar, M., Ishtiaq, F., Gupta, S. K., Martens, J., Harr, B., Singh, P. & Mohan, D. Niche filling slows the diversification of Himalayan songbirds. *Nature* **509**, 222–225 (2014).
3. Tucker, C. M., Cadotte, M. W., Carvalho, S. B., Davies, T. J., Ferrier, S., Fritz, S. A., Grenyer, R., Helmus, M. R., Jin, L. S., Mooers, A. O., Pavoine, S., Purschke, O., Redding, D. W., Rosauer, D. F., Winter, M. & Mazel, F. A guide to phylogenetic metrics for conservation, community ecology and macroecology. *Biological Reviews* **92,** 692–715 (2017).
4. Pavoine, S. & Ricotta, C. Functional and phylogenetic similarity among communities. *Methods Ecol Evol* **5,** 666–675 (2014).
5. Faith, D. P. Conservation evaluation and phylogenetic diversity. *Biological Conservation* **61,** 1–10 (1992).
