# Supplementary material for: Regional influences on community structure across the tropical-temperate divide
Source: Nat Commun. 2019 Jun 14;10:2646. doi: 10.1038/s41467-019-10253-6 (PMC6570764; doi:10.1038/s41467-019-10253-6)
Supplement: Supplementary file 2 — Reporting Summary [file 41467_2019_10253_MOESM2_ESM.pdf]

nature research

Corresponding author(s): Alexander E. White

Last updated by author(s): April 5, 2019

Reporting Summary

Nature Research wishes to improve the reproducibility of the work that we publish. This form provides structure for consistency and transparency in reporting. For further information on Nature Research policies, see Authors & Referees and the Editorial Policy Checklist.

Statistics

For all statistical analyses, confirm that the following items are present in the figure legend, table legend, main text, or Methods section.

n/a	Confirmed	
☐	☒	The exact sample size (*n*) for each experimental group/condition, given as a discrete number and unit of measurement
☐	☒	A statement on whether measurements were taken from distinct samples or whether the same sample was measured repeatedly
☐	☒	The statistical test(s) used AND whether they are one- or two-sided<br>*Only common tests should be described solely by name; describe more complex techniques in the Methods section.*
☐	☒	A description of all covariates tested
☐	☒	A description of any assumptions or corrections, such as tests of normality and adjustment for multiple comparisons
☐	☒	A full description of the statistical parameters including central tendency (e.g. means) or other basic estimates (e.g. regression coefficient) AND variation (e.g. standard deviation) or associated estimates of uncertainty (e.g. confidence intervals)
☐	☒	For null hypothesis testing, the test statistic (e.g. *F*, *t*, *r*) with confidence intervals, effect sizes, degrees of freedom and *P* value noted<br>*Give P values as exact values whenever suitable.*
☐	☒	For Bayesian analysis, information on the choice of priors and Markov chain Monte Carlo settings
☐	☒	For hierarchical and complex designs, identification of the appropriate level for tests and full reporting of outcomes
☒	☐	Estimates of effect sizes (e.g. Cohen's *d*, Pearson's *r*), indicating how they were calculated

Our web collection on statistics for biologists contains articles on many of the points above.

Software and code

Policy information about availability of computer code

Data collection	No software was used.
Data analysis	Data was analyzed using the ecostructure package in R. ecostructure contains a number of functions that make use of other open source packages in R. In particular, the model fits we report rely the R packages maptpx and CountClust.

For manuscripts utilizing custom algorithms or software that are central to the research but not yet described in published literature, software must be made available to editors/reviewers. We strongly encourage code deposition in a community repository (e.g. GitHub). See the Nature Research guidelines for submitting code & software for further information.

Data

Policy information about availability of data

All manuscripts must include a data availability statement. This statement should provide the following information, where applicable:

- Accession codes, unique identifiers, or web links for publicly available datasets
- A list of figures that have associated raw data
- A description of any restrictions on data availability

Local community bird surveys (species abundances), and the phylogeny of all birds included in the study are all publicly available as an ExpressionSet object in the R package ecostructure. Species abundances are also provided in Supplementary Data. Species range polygons are available upon request from BirdLife International at http://datazone.birdlife.org/species/requestdis.

Field-specific reporting

Please select the one below that is the best fit for your research. If you are not sure, read the appropriate sections before making your selection.

☐ Life sciences ☐ Behavioural & social sciences ☒ Ecological, evolutionary & environmental sciences

For a reference copy of the document with all sections, see nature.com/documents/nr-reporting-summary-flat.pdf

Ecological, evolutionary & environmental sciences study design

All studies must disclose on these points even when the disclosure is negative.

Study description	Using breeding bird distributions from the BirdLife International database in combination with a field studies of local Himalayan bird communities, we fit Grade of Membership models to examine compositional structure and turnover in bird species across the tropical-temperate climatic divide. Field studies were carried out at 38 sites across the Himalaya located between 150-4100 m, and for the global dataset, bird species were evaluated as present or absent in the terrestrial grid cells of a 1 x 1 degree raster of the globe.
Research sample	We evaluated breeding bird distributions for all birds globally using a publicly available dataset of GIS polygons. This dataset is available by request from BirdLife International. 9,518 species were included in the final analysis after accounting to terrestrial presence (present in a cell with >10% land cover).<br><br>Field surveys included abundances of all species of birds in 11 orders (Passeriformes, Piciformes, Bucerotiformes, Upupiformes, Trogoniformes, Coraciiformes, Cuculiformes, Psittaciformes, Apodiformes, Strigiformes and Columbiformes).
Sampling strategy	We attempted to survey every 500 m across the elevational gradients in the east and west which would give us complete transects. We added additional sites whenever possible to evaluate local species turnover. We conducted field surveys with two independent researchers.
Data collection	T.D.P. and D.M., plus up to two other experienced workers, independently censused birds in 38 5-hectare grids spanning the elevational gradient. In each grid two mornings were spent territory-mapping males, largely following the British Trust for Ornithology's common bird census protocols.
Timing and spatial scale	Presence-absence of global birds was determined using a 1 X 1 degree raster grid. Presences were determined by the overlap of each species breeding distribution with a given raster cell.<br><br>Field sites were located across the Himalaya
Data exclusions	Map cells that were ultimately included for analysis were those contained more than 3 species counted as present in the cell and those that overlapped land by at least 10%. These exclusion criteria were pre-established.<br><br>No field data were excluded.
Reproducibility	Findings include maximum likelihood model fits, not experimental treatments. However, we share all of the model fits and an R package to replicate the analysis.
Randomization	Our finding that species in our study fell into structured into groups was entirely generated by the model fits to the data. No prior assignation of the data to specific groups was involved in our study, except for the random initialization of our model.
Blinding	Blinding was not relevant to the study, as there were no experimental treatments.

Did the study involve field work? ☒ Yes ☐ No

Field work, collection and transport

Field conditions	Conditions ranged from tropical rainforest to freezing snowfields and included encounters with elephants, leopards, leeches and ticks.
Location	Locations were across the Himalaya. Site specific meta-data including latitude, longitude, and elevation is given in Supplementary Table 1.
Access and import/export	More than 50 permits were obtained for this study by coauthor Dhananjai Mohan, a professor at the Wildlife Institute of India. The Wildlife Institute of India has a government sanctioned Memorandum of Understanding (MoU) with the University of Chicago, which states that all permits will be obtained for the relevant research. We can provide a copy of the MoU upon request. No samples were collected for this study.
Disturbance	No disturbance. This work was conducted in collaboration with the Wildlife Institute of India, with full government and state permits.

Reporting for specific materials, systems and methods

We require information from authors about some types of materials, experimental systems and methods used in many studies. Here, indicate whether each material, system or method listed is relevant to your study. If you are not sure if a list item applies to your research, read the appropriate section before selecting a response.

Materials & experimental systems

n/a	Involved in the study
☒	☐ Antibodies
☒	☐ Eukaryotic cell lines
☒	☐ Palaeontology
☐	☒ Animals and other organisms
☒	☐ Human research participants
☒	☐ Clinical data

Methods

n/a	Involved in the study
☒	☐ ChIP-seq
☒	☐ Flow cytometry
☒	☐ MRI-based neuroimaging

Animals and other organisms

Policy information about studies involving animals; ARRIVE guidelines recommended for reporting animal research

Laboratory animals	The study did not involve laboratory animals.
Wild animals	Adult individuals from 304 bird species were observed and counted in their natural habitat across the Himalaya.
Field-collected samples	The study did not involve sampled collected in the field.
Ethics oversight	More than 50 permits were obtained for this study by coauthor Dhananjai Mohan, a professor at the Wildlife Institute of India. The Wildlife Institute of India has a government sanctioned Memorandum of Understanding (MoU) with the University of Chicago, which states that all permits will be obtained for the relevant research. We can provide a copy of the MoU upon request. No samples were collected for this study.

Note that full information on the approval of the study protocol must also be provided in the manuscript.
