# Supplementary material for: Regional influences on community structure across the tropical-temperate divide
Source: Nat Commun. 2019 Jun 14;10:2646. doi: 10.1038/s41467-019-10253-6 (PMC6570764; doi:10.1038/s41467-019-10253-6)
Supplement: Supplementary file 3 — Description of Additional Supplementary Files [file 41467_2019_10253_MOESM3_ESM.docx]

Description of Additional Supplementary Files

Supplementary Data 1 – Species contributions to Himalayan species abundance motifs Supplementary Data 2 – Species abundances in local Himalayan communities
